# Pre-Warning for the Remaining Time to Alarm Based on Variation Rates and Mixture Entropies

**DOI:** 10.3390/e27070736

**Published:** 2025-07-09

**Authors:** Zijiang Yang, Jiandong Wang, Honghai Li, Song Gao

**Affiliations:** 1 College of Electrical Engineering and Automation, Shandong University of Science and Technology, Qingdao 266590, China; zijiang_yang@sdust.edu.cn; 2 Shandong Luruan Digital Technology Co., Ltd., Jinan 250098, China; 15664408881@163.com; 3 Power Grid Center, Shandong Electric Power Research Institute for State Grid Corporation of China, Jinan 250000, China; songgao@yjy.sd.sgcc.com.cn

**Keywords:** variation rates, mixture entropy, upward trend, optimal pre-warning threshold, Bayesian estimation, remaining time to alarm

## Abstract

Alarm systems play crucial roles in industrial process safety. To support tackling the accident that is about to occur after an alarm, a pre-warning method is proposed for a special class of industrial process variables to alert operators about the remaining time to alarm. The main idea of the proposed method is to estimate the remaining time to alarm based on variation rates and mixture entropies of qualitative trends in univariate variables. If the remaining time to alarm is no longer than the pre-warning threshold and its mixture entropy is small enough then a warning is generated to alert the operators. One challenge for the proposed method is how to determine an optimal pre-warning threshold by considering the uncertainties induced by the sample distribution of the remaining time to alarm, subject to the constraint of the required false warning rate. This challenge is addressed by utilizing Bayesian estimation theory to estimate the confidence intervals for all candidates of the pre-warning threshold, and the optimal one is selected as the one whose upper bound of the confidence interval is nearest to the required false warning rate. Another challenge is how to measure the possibility of the current trend segment increasing to the alarm threshold, and this challenge is overcome by adopting the mixture entropy as a possibility measurement. Numerical and industrial examples illustrate the effectiveness of the proposed method and the advantages of the proposed method over the existing methods.

## 1. Introduction

Alarm systems are paramount to the safety of industrial processes [1,2] and are integrated into distributed control systems as their essential parts. With the complexity of industrial processes increasing, thousands of process variables are required to be monitored intelligently. Alarm systems monitor abnormalities in industrial processes automatically by comparing the amplitudes of process variables with their alarm thresholds. When the amplitude of one process variable is larger (or smaller) than its high (or low) alarm threshold, an alarm is triggered to notify the operators of an abnormality occurring, and then the operators take effective actions to restore the industrial process to its normal situation as soon as possible [3]. Research topics about alarm systems have been attracting attention from industrial organizations and academic societies for decades [1,2,4,5,6,7], and a large number of existing publications about alarm systems focus on alarm threshold optimization [8,9,10], nuisance alarm suppression [11,12], and alarm root cause analysis [13,14,15]. In addition to the research topics aforementioned, the pre-warning (or early warning) design is also a popular research topic about alarm systems.

Pre-warnings are indispensable for a special class of industrial process variables. Once alarms are triggered for these process variables, accidents with negative effects will occur. This phenomenon is referred to as alarms being accidents [16]. An essential reason for such a phenomenon is that there is too little time left for industrial plant operators to handle the occurring alarms and to take actions to avoid the upcoming accidents. Therefore, pre-warnings need to be designed to inform industrial plant operators about the remaining time of these process variables reaching their alarm thresholds.

Pre-warning-related research has been ongoing for decades [17,18], and the existing methods can be divided into univariate data-driven methods and multivariate data-driven methods [19,20]. Although the univariate data-driven methods are the origin of pre-warning methods, the associated studies are rather limited. Qu et al. [21] explored pre-warnings for pipeline leakage detection by analyzing the vibration signals through wavelet packet decomposition and support vector machine. Jiang et al. [22] established a complete ensemble empirical mode decomposition with adaptive noise to obtain components associated with early warnings, and they used decision tree and support vector machine to classify normal and abnormal states to generate early warnings. Zhang et al. [23] investigated an adaptive pre-warning method based on trend monitoring for an oil refining process, by checking if the process variables were steady or not. Jin et al. [24] formed an early fault warning method for thermal equipment by using incremental Gaussian mixture regression. He et al. [25] advocated a support vector machine ensemble model construction method to enhance the effectiveness of early warning, and they validated the method through wind turbine data and UCI benchmark datasets. Wang et al. [26] extracted cavitation features through a multi-index fusion-based method to formulate pre-warnings according to the $T^{2}$ test in the hydraulic turbine cavitation detection. Cheng et al. [27] generated early warnings for charging the thermal runaway of electric vehicle lithium-ion batteries, based on the residuals between a long- and short-term memory network and a temporal convolutional network predicting charging temperature and real charging data.

Multivariate data-driven methods have attracted much more attention. Cai et al. [28] predicted alarm events through a long short-term memory network and the Word2Vec of the natural language processing approach based on the alarm log. Geng et al. [29] proposed an intelligent early-warning method based on moving window sparse principal component analysis for abnormal detection in chemical processes. Sun et al. [30] executed pre-warning for a dry-type transformer through a temperature-based model established by the sparse Bayesian learning algorithm. Arunthavanathan et al. [31] formulated a convolutional neural network–long short-term memory network-based model for early fault detection and prognosis in multivariate process systems. Mamudu et al. [32] integrated a multilayer perceptron–artificial neural network model and a Bayesian network to offer pre-warnings in a hydrocarbon production system. Kopbayev et al. [33] performed gas leakage early detection through a convolutional network combined with bi-directional long short-term memory layer network model trained with image data. He et al. [34] presented an improved TOLOv3 algorithm to formulate a recognition and pre-warning system for tank leaks. Han et al. [35] advocated a dynamic uncertain causality graph-based method to identify the root fault cause of regenerative thermal oxidizers by incorporating expert knowledge. Song et al. [36] formulated a target detection model based on an image processing hierarchical algorithm to warn rust in transmission line connection fittings. Ali et al. [37] proposed a wavelet entropy-based multi-scale PCA–SDG methodology for industrial process monitoring. Fu et al. [38] investigated a multi-scale entropy-based feature extraction method to assign warnings for the compressor instability inception.

The limitations of the existing univariate data-driven methods lie in the facts that they do not consider the variation rates of process variables raising (decreasing) to their alarm thresholds and that they are not able to obtain the remaining time to alarm as important information for pre-warnings. The main purpose of this paper is to generate pre-warnings to inform operators about the remaining time of the process variables reaching their alarm thresholds. There are two challenges to obtaining the desired pre-warnings. First, the pre-warning threshold is determined from the historical data sequences in normal situations to meet with a required false warning rate. Second, the uncertainty of the raising (decreasing) trend should be measured mathematically in order to tell whether the pre-warnings are reliable.

A data-driven pre-warning method is proposed in this paper for the special class of univariate process variables. By extracting the last data samples, the variation rates, and the time durations of qualitative trends through the piecewise linear representation (PLR) method from a historical data sequence, the optimal pre-warning threshold is determined through a sample distribution of the remaining time to alarm, which is calculated with the obtained variation rates and the last data samples of the upward trend segments. In addition, sample distributions of the last data samples, the variation rates, and the time durations of the trend segments are obtained. For a current trend segment online, the entropies of its first data sample, variation rate, and time duration are summed with weighting factors to form a mixture entropy, and its remaining time to alarm is estimated with its variation rate and last data sample. If the remaining time to alarm is no longer than the optimal pre-warning threshold and the mixture entropy is small enough then a pre-warning is generated.

The pre-warning referred to in this paper has a different indication to those in the existing literature. The pre-warning indicates timely warnings in most of the existing literature. That is, the existing pre-warning methods define pre-warnings as having a short detection time, which is the time interval between the instants of the abnormality occurring and the warning. Although the existing methods are applied successfully in their scenarios, they do not take the variation rates to formulate predictions and do not take the remaining time to alarm as the features to formulate pre-warnings. In other words, these pre-warnings cannot indicate the remaining time to alarm, due to the fact that the variation rates of the process variables are varying. As a result, the existing methods cannot be used to generate the desired pre-warnings for the special class of industrial process variables being considered in this context.

The rest of this paper is organized as follows: Section 2 describes the problem to be solved. Section 3 presents the detailed steps of the proposed method in three subsections. Section 4 provides numerical and industrial examples to illustrate the effectiveness of the proposed method. Section 5 concludes the paper.

## 2. Problem Description

Given a univariate process variable *x* configured with a high alarm threshold $x_{th}$ and that x(n) is sampled from *x* in normal situations with a sampling period of *h* (e.g., *h* = 1 s), let ${\left\{x\left(n\right)\right\}}_{n=1}^{N}$ be a historical data sequence of *x*. The alarm data sequence ${\left\{x_{a}\left(n\right)\right\}}_{n=1}^{N}$ corresponding to ${\left\{x\left(n\right)\right\}}_{n=1}^{N}$ is generated as

(1) $$x_{a}\left(n\right)=\left\{\begin{array}{cc} 1, & \mathrm{if}\;x\left(n\right)\geq x_{th}\\ 0, & \mathrm{otherwise} \end{array}\right..$$

**Figure 1:**
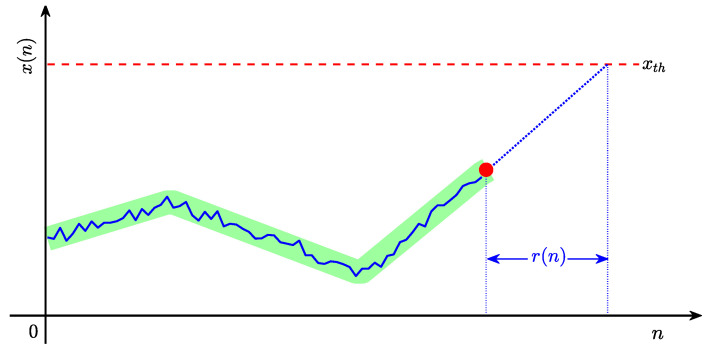
The illustration diagram of trend segments and the remaining time to alarm. The alarm threshold $x_{th}$ (red dashed), the sequence of x(n) (blue solid), the trend of *x* (light green), and the current sample of x(n) (red point).

Suppose that *x* is one of the industrial process variables considered in this paper, and an alarm in *x* indicates an accident. Hence, a pre-warning for the remaining time to alarm is necessary. The remaining time to alarm is defined as the time span over which *x* increases from the current sample x(n) to its alarm threshold $x_{th}$. To illustrate this definition clearly, a diagram is provided in Figure 1, where the blue solid curve represents a sequence of x(n) and the red dashed line is the high alarm threshold $x_{th}$. As depicted in Figure 1, the sequence of x(n) involves three trend segments, which are covered by light-green bars and can reflect the real changes in *x*. The first segment shows an upward trend, the second segment shows a downward trend, and the third segment involving the current sample x(n) indicates an upward trend. The current sample is marked with the red point in Figure 1. If x(n) is believed to arrive at $x_{th}$ with the variation rate of the current trend then the remaining time to alarm r(n) can be regarded as the time span between the current sample index *n* and the instant of x(n) arriving at $x_{th}$, marked by the two vertical blue dotted lines. Hence, r(n) can be calculated as

(2) $$r\left(n\right)=\frac{x_{th}-x\left(n\right)}{v(n)},$$

where v(n) is the variation rate of the current trend, and where v(n) is equal to the trend amplitude changing within a fixed time interval:

(3) $$v\left(n\right)\;=\;\frac{x\left(n_{2}\right)\;-\;x\left(n_{1}\right)}{n_{2}\;-\;n_{1}},n_{2}>n_{1}.$$

Here, $x(n_{1})$ and $x(n_{2})$ are data samples contained in the current trend; $n_{1}$ and $n_{2}$ are the sampling indices of $x(n_{1})$ and $x(n_{2})$, respectively.

To determine a threshold for the remaining time to alarm, r(n), an assumption is required that the sample distribution of r(n) does not change. This assumption is reasonable, due to the fact that the variation rates of process variables in normal situations are in certain intervals, and this fact is supported by physical balances in industrial practice, such as material balance and energy balance. Additionally, if r(n) is regarded as a random variable, a pre-warning threshold $r_{th}$ can be determined from the theoretical probability distribution of r(n) by considering a required false warning rate $f_{0}$ as

(4) $$p\left(r\left(n\right)\leq r_{th}\right)=f_{0},$$

where $p(r\left(n\right)\leq r_{th})$ denotes the theoretical probability of r(n) no more than the pre-warning threshold $r_{th}$. Eventually, a pre-warning data sequence $r_{w}\left(n\right)$ can be generated online as

(5) $$r_{w}\left(n\right)=\left\{\begin{array}{cc} 1, & \mathrm{if}\;r\left(n\right)\leq r_{th}\\ 0, & \mathrm{otherwise} \end{array}\right..$$

The objective of this paper was to determine an optimal pre-warning threshold $r_{th}$ with the constraint of the required false warning rate $f_{0}$ and to obtain the reliable pre-warning sequence $r_{w}\left(n\right)$ in Equation (5). Three steps can be adopted to realize this objective. The first step is to extract the last data samples, the variation rates, and the time durations of the trend segments in x(n). The second step is to determine an optimal pre-warning threshold for the remaining time to alarm from its one sample distribution, which is obtained from the previously extracted information. The third step is to generate pre-warnings by comparing the online estimated remaining time to alarm with the optimal pre-warning threshold by considering the probability of the current trend arriving at the alarm threshold $x_{th}$.

**Figure 2:**
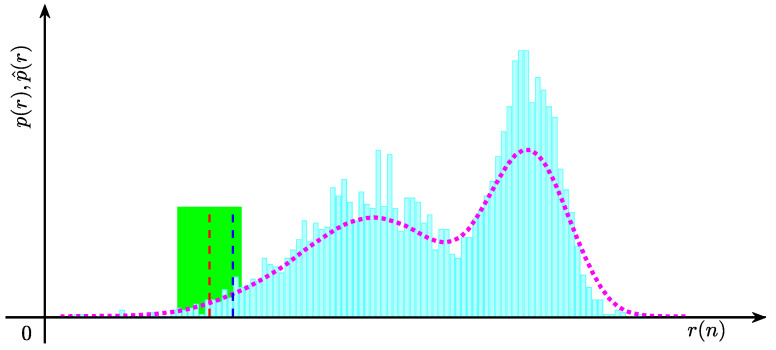
An illustration diagram of the challenge to be resolved. The sample distribution of r(n) (light-blue bars), the theoretical probability distribution of r(n) (magenta dashed), the theoretical value of the pre-warning threshold (blue dashed), an estimated pre-warning threshold (red dashed), and its uncertainties (rectangle with a green surface).

There are two challenges to obtaining the pre-warning sequence of the remaining time to alarm. First, as illustrated in Figure 2, an estimated probability distribution $\hat{p}\left(r\right)$ of the remaining time to alarm r(n) obtained from a sample distribution (light-blue bars) is always an approximation of the theoretical distribution p(r) (magenta dashed curve). This approximation leads to uncertainties (light-red rectangle) in the pre-warning threshold (red dashed line) determined from $\hat{p}\left(r\right)$ for a given $f_{0}$ and induces a deviation between the theoretical pre-warning threshold (blue dashed line) and the determined pre-warning threshold. If a large part of the uncertainties is located on the right side of the theoretical pre-warning threshold then the determined pre-warning threshold might result in many false pre-warnings. Therefore, the uncertainties should be considered in determining the optimal pre-warning threshold. Second, as shown in Figure 1, the current trend is not bound to increase to the alarm threshold, and it might change its direction randomly in the following time. Additionally, disturbances involved in x(n) might have large variation rates, which could result in small r(n)s in Equation (2) and make $r_{w}\left(n\right)$s be 1 in Equation (5). Therefore, it is necessary to measure the possibility of the current trend increasing to the alarm threshold.

## 3. The Proposed Method

This section presents the detailed steps of the proposed method in three subsections and makes a summary for the proposed method in the fourth subsection.

### 3.1. Determining the Optimal Pre-Warning Threshold

The method for determining an optimal pre-warning threshold with the constraint of the false pre-warning rate $f_{0}$ is presented here. Suppose that the last data sample $x_{lp}\left(k\right)$ of the *k*-th trend segment and its variation rate v(k) are obtained from ${\left\{x\left(n\right)\right\}}_{n=1}^{N}$, 1≤k≤K. Here, *K* denotes the number of the trend segments involved in ${\left\{x\left(n\right)\right\}}_{n=1}^{N}$. The remaining time to alarm cannot be calculated according to Equation (2) directly, due to the fact that $x_{lp}\left(k\right)$ may be larger than $x_{th}$ when the historical data sequence corresponds to abnormal conditions. If $x_{lp}\left(k\right)\geq x_{th}$ then the remaining time to alarm r(k) takes a negative value, which is contrary to the meaning of r(k). Consequently, Equation (2) is rewritten as

(6) $$r\left(k\right)\;=\;\left\{\begin{array}{cc} \left\lceil \frac{x_{th}-{\hat{x}}_{lp}\left(k\right)}{v(k)}\right\rceil , & \mathrm{if}\;{\hat{x}}_{lp}\left(k\right)\;<\;x_{th}\;\mathrm{and}\;v\left(k\right)\geq v_{k,th}\\ C_{T}, & \mathrm{if}\;{\hat{x}}_{lp}(k)\;<\;x_{th}\;\mathrm{and}\;v\left(k\right)<v_{k,th}\\ 0, & \mathrm{if}\;{\hat{x}}_{lp}\left(k\right)\;\geq \;x_{th} \end{array}\right.,$$

where ${\hat{x}}_{lp}\left(k\right)$ is an approximation of $x_{lp}\left(k\right)$ to reduce the effect of noise, $v_{k,th}$ is a significant threshold of variation rates, and $v\left(k\right)\geq v_{k,th}$ indicates a significant upward trend existing. The operator ⌈·⌉ takes the smallest integer more than the operand; $C_{T}$ denotes a large constant specified by users, that is, r(k) takes a constant value $C_{T}$ when $v\left(k\right)\leq v_{k,th}$ and ${\hat{x}}_{lp}\left(k\right)<x_{th}$. The methods used to determine the value of v(k) and $v_{k,th}$ are introduced in the next subsection.

If the theoretical probability distribution of the remaining time to alarm is available then an optimal pre-warning threshold of r(k) can be determined by taking the required false pre-warning rate $f_{0}$ into consideration as

(7) $$r_{th,opt}=\arg\min_{r_{th}}\left|P\left(r\left(k\right)\leq r_{th}\right)-f_{0}\right|.$$

Here, $P(r\left(k\right)\leq r_{th})$ is the theoretical false warning rate of r(k) being no more than $r_{th}$. Unfortunately, the theoretical probability distribution of the remaining time to alarm cannot be provided. Hence, an alternative strategy of determining the optimal pre-warning threshold is to take advantage of a sample distribution of r(k). With a sample distribution of r(k), $P(r\left(k\right)\leq r_{th})$ can be approximated well by the false warning rate $f(r_{th})$ when the number of r(k) in the sample distribution tends to infinity, that is,

(8) $$f\left(r_{th}\right)\overset{k\to +\infty }{=}P\left(r\left(k\right)\leq r_{th}\right).$$

Due to the number of r(k) in a sample distribution always being finite, uncertainties induced by the approximation exist in all determined $r_{th}$s. Thereby, even if a pre-warning threshold $r_{th}$ is selected as the one that satisfies Equation (7) the actual false warning rate might be larger than $f_{0}$. Hence, a conservative value of $r_{th}$ is selected as $r_{th,opt}$, which corresponds to the (1−α)% confidence interval upper bound ${\bar{f}}_{\alpha }\left(r_{th}\right)$ of $f(r_{th})$, that is,

(9) $$r_{th,opt}=\arg\min_{r_{th}}\left|{\bar{f}}_{\alpha }\left(r_{th}\right)-f_{0}\right|.$$

The value of ${\bar{f}}_{\alpha }\left(r_{th}\right)$ can be determined through Theorem 1:

**Theorem 1.**  *Suppose that there are M samples contained in a sample distribution of r(m). For a given $r_{th}$, the (1−α)% confidence interval upper bound ${\bar{f}}_{\alpha }\left(r_{th}\right)$ of $f(r_{th})$ satisfies*

(10) $$\int_{-\infty }^{{\bar{f}}_{\alpha }\left(th\right)}\;\;\;\frac{p_{F|M_{a}}\left(f\left(r_{th}\right)|M_{a}\right)}{\int_{0}^{1}p_{F|M_{a}}\left(f\left(r_{th}\right)|M_{a}\right)df\left(r_{th}\right)}=\;\;1\;-\;\alpha /2,$$

*where $p_{F|M_{a}}\left(f\left(r_{th}\right)|M_{a}\right)$ is a posterior probability mass function of $f(r_{th})$,*

$$p_{F|M_{a}}\left(f|M_{a}\right)\;=\;\;\frac{M_{a}!f^{M_{a}}\left(r_{th}\right){\left(1\;-\;f\left(r_{th}\right)\right)}^{M\;-\;M_{a}}}{\sqrt{2\pi }M_{a}!\left(M\;-\;M_{a}\right)e^{-72{\left(f\left(r_{th}\right)-\;1/2\right)}^{2}\;}}.$$

*Here, $M_{a}$ is the number of r(m) satisfying $r\left(m\right)\leq r_{th}$; M and $M_{a}$ are random variables whose realizations are M and $M_{a}$, respectively.*

**Proof of Theorem 1.**  If *M* and $M_{a}$ are regarded as realizations of random variables M and $M_{a}$, respectively, then f (a simple expression of $f(r_{th})$) can be regarded as a realization of the false warning rate F. Due to the fact that r(m)s are independent of each other, $M_{a}$ follows a binomial distribution for a given threshold $r_{th}$ [39]. Therefore, the conditional probability mass function of $M_{a}$ based on f can be calculated as

(11) $$p_{M_{a}|F}\left(M_{a}|f\right)\;=\;\frac{M_{a}!f^{M_{a}}{\left(1\;-\;f\right)}^{M\;-\;M_{a}}}{M_{a}!\left(M\;-\;M_{a}\right)}.$$

Because there is no available knowledge about F and f takes a value in (0,1), a reasonable choice is to take the prior probability mass function of f as a normal distribution with mean $\mu_{f}$ and standard variance $\sigma_{f}$ [40,41], i.e.,

(12) $$p_{F}\left(f\right)=\left\{\begin{array}{c} \frac{1}{\sqrt{2\pi }}e^{-\frac{{\left(f-\mu_{f}\right)}^{2}}{2\sigma_{f}^{2}}},\;\mathrm{if}\;0<f<1.\\ 0,\;\mathrm{otherwise}. \end{array}\right.$$

Based on Equations (11) and (12), the joint probability mass function of $M_{a}$ and f is

(13) $$\begin{array}{c} p_{M_{a},F}\left(M_{a},f\right)=p_{M_{a}|F}\left(M_{a}|f\right)\cdot p_{F}\left(f\right)\\ =\;\frac{1}{\sqrt{2\pi }}\;\frac{M_{a}!f^{M_{a}}{\left(1\;\;-\;\;f\right)}^{M\;-\;M_{a}}}{M_{a}!\left(M\;\;-\;\;M_{a}\right)}e^{-\;\frac{{\left(f\;-\;\mu_{f}\right)}^{2}}{2\sigma_{f}^{2}}}. \end{array}$$

According to the Bayesian formula, the posterior probability mass function of f based on the realization $M_{a}$ of $M_{a}$ is

(14) $$p_{F|M_{a}}\left(f|M_{a}\right)=\frac{p_{M_{a},F}\left(M_{a},f\right)}{\int_{0}^{1}p_{M_{a},F}\left(M_{a},f\right)df}.$$

By taking Equation (11) into Equation (14), it can be obtained that

(15) $$p_{F|M_{a}}\left(f|M_{a}\right)\;\;=\;\;\frac{\frac{1}{\sqrt{2\pi }}\;\frac{M_{a}!f^{M_{a}}{\left(1\;-\;f\right)}^{M-M_{a}}}{M_{a}!\left(M-M_{a}\right)}e^{-\;\frac{{\left(f\;-\;\mu_{f}\right)}^{2}}{2\sigma_{f}^{2}}}}{\int_{0}^{1}\;\;\frac{1}{\sqrt{2\pi }}\;\frac{M_{a}!f^{M_{a}}{\left(1\;-\;f\right)}^{M-M_{a}}}{M_{a}!\left(M-M_{a}\right)}e^{-\;\frac{{\left(f\;-\;\mu_{f}\right)}^{2}}{2\sigma_{f}^{2}}}df}.$$

With the posterior probability mass function of f in Equation (15), the (1−α)% confidence interval upper bound ${\bar{f}}_{\alpha }$ of the estimate about f satisfies

(16) $$\int_{-\infty }^{\bar{f_{\alpha }}}p_{F|M_{a}}\left(f|M_{a}\right)df=1-\alpha /2.$$

According to the three-sigma rule of thumb, it is common to choose $\mu_{f}=1/4$ and $\sigma_{f}=1/12$ in Equation (12). By taking Equation (15) into Equation (16), we are ready to obtain Equation (10).    □

### 3.2. Extracting Features from an Historical Data Sequence

The last data samples, the variation rates, and the time durations of the trend segments contained in ${\left\{x\left(n\right)\right\}}_{n=1}^{N}$ are extracted through a piecewise linear representation (PLR) method to support the formulation of the proposed method. The PLR method used here is an improved version of the sliding window and bottom-up method [42]. By considering the last data samples $x_{lp}\left(k\right)$s and variation rates v(k)s used in Equation (6) and the duration times d(k)s used in the next subsection, these features of the trend segments are extracted at the same time.

The main idea of the PLR method is to approximate a data sequence with straight line segments. By pushing the historical data samples of ${\left\{x\left(n\right)\right\}}_{n=1}^{N}$ into a buffer one by one, a data segment ${\left\{x_{0}\left(l\right)\right\}}_{l=1}^{L}$ is contained in the buffer. Here, *L* is the number of data samples contained in it, $L\in Z^{+}$, and *L* is no more than the buffer size *W*. The buffer size *W* is crucially important for obtaining the variation rates and the sample distribution of the remaining time to alarm. The value of *W* is selected as double the maximum length of the trend segments, which are obtained from a test data sequence and can reflect the trends of the test data sequence effectively. The first and latest data samples in the buffer are $x_{0}\left(1\right)$ and $x_{0}\left(L\right)$. If ${\left\{x_{0}\left(l\right)\right\}}_{l=1}^{L}$ can be approximated by one straight line segment then the PLR of ${\left\{x_{0}\left(l\right)\right\}}_{l=1}^{L}$ is expressed as

(17) $${\hat{x}}_{0}\left(l\right)={\hat{b}}_{0}\cdot l+{\hat{a}}_{0},l=1,2,\ldots ,L.$$

Here, the parameters ${\hat{a}}_{0}$ and ${\hat{b}}_{0}$ are obtained by minimizing the sum of squared errors between $x_{0}\left(l\right)$ and ${\hat{x}}_{0}\left(l\right)$, i.e.,

(18) $$\left({\hat{a}}_{0},{\hat{b}}_{0}\right)=\arg\min_{a_{0},b_{0}}\sum_{l=1}^{L}{\left(x_{0}\left(l\right)-{\hat{a}}_{0}-{\hat{b}}_{0}\cdot l\right)}^{2}.$$

The parameters ${\hat{b}}_{0}$ and ${\hat{a}}_{0}$ are estimated analytically as [43]

(19) $${\hat{b}}_{0}=\frac{\sum_{l=1}^{L}l\cdot x_{0}\left(l\right)-\sum_{l=1}^{L}l\cdot \sum_{l=1}^{L}x_{0}\left(l\right)}{L\cdot \sum_{l=1}^{L}l^{2}-{\left(\sum_{l=1}^{L}l\right)}^{2}},$$

(20) $${\hat{a}}_{0}={\bar{x}}_{0}\left(l\right)-{\hat{b}}_{0}\cdot \bar{l},$$

where ${\bar{x}}_{0}\left(l\right)=\sum_{l=1}^{L}x_{0}\left(l\right)/L$, $\bar{l}=\sum_{l=1}^{L}l/L$. Whether the data segment in the buffer can be represented by a straight line segment in Equation (17) or not is determined as

(21) $$\left\{\begin{array}{cc} \varepsilon \left(l\right)<\varepsilon_{0}, & \mathrm{if}\;\forall l\in [1,L]\\ \varepsilon \left(l\right)\geq \varepsilon_{0}, & \mathrm{if}\;\exists l\in [1,L+1] \end{array}\right.,$$

where $\varepsilon_{0}$ is a separation threshold associated with the variance of noise contained in x(n). A default value of $\varepsilon_{0}$ can be determined as the significant change amplitude [44]. The variable ε(l) is the Euclidean distance of x(l) to the approximating straight line segment in Equation (17), i.e.,

(22) $$\varepsilon \left(l\right)=\frac{|{\hat{b}}_{0}\cdot l+{\hat{a}}_{0}-x_{0}\left(l\right)|}{\sqrt{{\hat{b}}_{0}^{2}+1}}.$$

If ε(l) is larger than $\varepsilon_{0}$ for ${\left\{x_{0}\left(l\right)\right\}}_{l=1}^{L+1}$, ∀l∈[1,L+1], and if all ε(l)s are smaller than $\varepsilon_{0}$ for ${\left\{x_{0}\left(l\right)\right\}}_{l=1}^{L}$, ∀l∈[1,L], then ${\left\{x_{0}\left(l\right)\right\}}_{l=1}^{L}$ is regarded as a new PLR segment and the buffer is emptied and updated as $x_{0}\left(1\right)=x_{0}\left(L+1\right)$. Suppose that there are *K* PLR segments obtained from the historical data sequence ${\left\{x\left(n\right)\right\}}_{n=1}^{N}$, $K\in Z^{+}$. To ease notations, the *k*-th PLR segment is denoted by ${\left\{x\left(n\right)\right\}}_{n=n_{k}}^{n_{k}+L_{k}}$, k∈[1,K], and its PLR result and PLR parameters are denoted by ${\left\{\hat{x}\left(n\right)\right\}}_{n=n_{k}}^{n_{k}+L_{k}}$, ${\hat{b}}_{k}$, ${\hat{a}}_{k}$, respectively. Here, $n_{k}$ and $L_{k}$ are the first sampling index and the data length of the *k*-th PLR segment.

According to the meaning of ${\hat{b}}_{0}$ in Equation (17), it is obvious that ${\hat{b}}_{k}$ is the variation rate of the *k*-th PLR segment ${\left\{\hat{x}\left(n\right)\right\}}_{n=n_{k}}^{n_{k}+L_{k}}$. To determine whether a trend segment with variation rate ${\hat{b}}_{k}$ is an upward trend, the significant threshold of variation rate $v_{m,th}$ in Equation (6) can be selected as the upper bound of the (1−β)% confidence interval of ${\hat{b}}_{k}$ [45] (page 41 therein),   

(23) $$v_{m,th}=\frac{t_{\left(L_{k}-2,\beta /2\right)}\cdot \sum_{n=n_{k}}^{n_{k}+L_{k}}{\left(x\left(n\right)-\hat{x}\left(n\right)\right)}^{2}}{\sqrt{\sum_{n=n_{k}}^{n_{k}+L_{k}}{\left(n-\bar{n}\right)}^{2}}}.$$

Here, $t_{\left(L_{k}-2,\beta /2\right)}$ is the (1−β/2)% percentile of the Student’s distribution with the freedom degree $\left(L_{k}-2\right)$ [45]. The default value of β is 0.05.

For the *k*-th PLR segment ${\left\{\hat{x}\left(n\right)\right\}}_{n=n_{k}}^{n_{k}+L_{k}}$, its last data sample and the duration time are

(24) $$\left\{\begin{array}{c} x_{lp}\left(k\right)=\hat{x}\left(n_{k}+L_{k}\right)\\ d\left(k\right)=L_{k} \end{array}\right..$$

Additionally, the variation rate of ${\left\{\hat{x}\left(n\right)\right\}}_{n=n_{k}}^{n_{k}+L_{k}}$ is

(25) $$v\left(k\right)={\hat{b}}_{k}.$$

When all the trend segments in ${\left\{x\left(n\right)\right\}}_{n=1}^{N}$ are obtained, we are ready to obtain three sample distributions corresponding to the variation rate v(k), the last data sample $x_{lp}\left(k\right)$, and the duration time d(k), separately. To simplify the representation of these sample distributions, the three sample distributions are denoted as $F_{lp}$, $F_{v}$, and $F_{d}$, respectively.

### 3.3. Generating Pre-Warnings by Combining the Mixture Entropies

Pre-warnings are generated with a combination of the online estimated remaining time to alarm and the mixture entropy of the current trend segment. To estimate the remaining time to alarm online, the PLR method introduced in Section 3.1 is adopted to obtain variation rates online. Suppose that the online data samples contained in the buffer are ${\left\{x_{ol}\left(l\right)\right\}}_{l=1}^{L_{ol}}$ and $\varepsilon \left(l\right)<\varepsilon_{0}$, $\forall l\in [1,L_{ol}]$, $L_{ol}\leq W$. Here, the parameter $L_{ol}$ is a variable that is increasing with the sampling time. The last component of ${\left\{x_{ol}\left(l\right)\right\}}_{l=1}^{L_{ol}}$ is the current data sample $x_{ol}\left(n\right)$, which is denoted by $x_{ol}\left(L_{ol}\right)$. By taking Equations (19), (21), and (25) into consideration, the approximation of $x_{ol}\left(l\right)$ is

(26) $${\hat{x}}_{ol}\left(l\right)\;=\;\left\{\begin{array}{cc} \sum_{l=1}^{L_{ol}}\left[l\cdot x_{ol}\left(l\right)-l\cdot \sum_{l=1}^{L_{ol}}x_{ol}\left(l\right)\right], & \mathrm{if}\;L_{ol}\;\geq \;L_{min}\\ {\hat{x}}_{ol}\left(n-L_{ol}-1\right), & \mathrm{if}\;L_{ol}\;<\;L_{min} \end{array}\right.,$$

where $L_{min}$ is the minimum required length of the data samples contained in the buffer; ${\hat{x}}_{ol}\left(n-L_{ol}-1\right)$ is the approximation of the last data sample in the previous adjacent PLR segment. With ${\hat{x}}_{ol}\left(l\right)$ in Equation (26), the variation rate of ${\left\{x_{ol}\left(l\right)\right\}}_{l=1}^{L_{ol}}$ is estimated as

(27) $$v_{ol}\left(n\right)\;=\;\left\{\begin{array}{cc} \frac{\sum_{l=1}^{L_{ol}}\left[l\cdot {\hat{x}}_{ol}\left(l\right)-l\cdot \sum_{l=1}^{L_{ol}}{\hat{x}}_{ol}\left(l\right)\right]}{L\cdot \sum_{l=1}^{L_{ol}}l^{2}-{\left(\sum_{l=1}^{L_{ol}}l\right)}^{2}}, & \mathrm{if}\;L_{ol}\;\geq \;L_{min}\\ v_{ol}\left(n-1\right), & \mathrm{if}\;L_{ol}\;<\;L_{min} \end{array}\right..$$

Here, $v_{ol}\left(n-1\right)$ is the variation rate of ${\hat{x}}_{ol}\left(n-1\right)$ obtained from the previous adjacent PLR segment. The lengths of the PLR segments are adjusted according to the different types of gradual or abrupt process situation changes. According to Equations (21), (26), and (27), the separation threshold $\varepsilon_{0}$ and the minimum required length of data samples $L_{min}$ can assure the segment length varying in different situations. For a gradual change, its corresponding variables vary smoothly, and the variable ε(l) in Equation (22) might be smaller than $\varepsilon_{0}$ for a large time interval, and then a large segment length can be obtained. Conversely, if there is an abrupt change, the variables vary dramatically so that ε(l) in Equation (22) might be larger than $\varepsilon_{0}$ in a short time interval (no less than $L_{min}$), and a short segment is obtained. The minimum required length of data samples, $L_{min}$, is used to avoid wrong segmentation caused by outliers or disturbances in data sequences and to avoid inaccurate variation rates obtained from PLR. In other words, the minimum required length of data samples, $L_{min}$, assures the data segments being trends.

Because there is no prior information about the upcoming data samples after $x_{ol}\left(n\right)$, a conservative assumption is that $x_{ol}\left(n\right)$ is the last data sample of the current trend. Hence, the online alarm remaining time can be predicated with ${\hat{x}}_{ol}\left(n\right)$ and $v_{ol}\left(n\right)$ as

(28) $$r_{ol}\left(n\right)\;=\;\left\{\begin{array}{cc} \left\lceil \frac{x_{th}-{\hat{x}}_{ol}\left(n\right)}{v_{ol}\left(n\right)}\right\rceil , & \mathrm{if}\;{\hat{x}}_{ol}\left(n\right)\;<\;x_{th}\;\mathrm{and}\;v_{ol}\left(n\right)\;\geq \;v_{th}\\ C_{T}, & \mathrm{if}\;{\hat{x}}_{ol}\left(n\right)\;<\;x_{th}\;\mathrm{and}\;v_{ol}\left(n\right)\;<\;v_{th}\\ 0, & \mathrm{if}\;{\hat{x}}_{ol}\left(n\right)\;\geq \;x_{th} \end{array}\right..$$

Here, $x_{th}$ is the high alarm threshold of *x* in Equation (1), the operator ⌈·⌉ takes the smallest integer more than the operand, and $v_{th}$ is the upper bound of the (1−α)% confidence interval of $v_{ol}\left(n\right)$. The value of $v_{th}$ is determined with ${\left\{x_{ol}\left(l\right)\right\}}_{l=1}^{L_{ol}}$ through Equation (23).

Although the optimal pre-warning threshold $r_{th,opt}$ is determined in Equation (9), noise and disturbances involved in *x* could lead to several $v_{ol}\left(n\right)$s in Equation (28) with a large positive value and let the corresponding $r_{ol}\left(n\right)$s be less than the optimal pre-warning threshold $r_{th,opt}$. In addition, it can be concluded from Equation (28) that the trend segments tending to arrive at the alarm threshold should be in one of the following two cases: (1) the trend segment has had a large positive variation rate for a long time; (2) the trend segment starts at a large amplitude value and with a large positive variation rate. According to the information entropy theory, an entropy with a small value indicates a higher certainty [46]. To measure the certainty of trend segments tending to arrive at the alarm threshold, the information entropy as well as the conditional entropy would be the right candidates. Otherwise, by considering the two cases mentioned above, variation rates, variation time, and amplitude value are all the critical factors for determining if the trend segment arrives at the alarm threshold or not, and they are independent of each other as observed in industrial data sequences. As a result, the information entropy and the conditional entropy cannot be used here, and a mixture entropy is designed to measure the certainty of the estimated remaining time to alarm as

(29) $$H\left(x_{ol}\left(n\right)\right)=\omega_{1}\cdot H_{lp}\left({\hat{x}}_{ol}\left(n\right)\right)+\omega_{2}\cdot H_{v}\left(v_{ol}\left(n\right)\right)+\omega_{3}\cdot H_{d}\left(L_{ol}\right),$$

where $\omega_{1}$, $\omega_{2}$, and $\omega_{3}$ are weighted parameters, $\sum_{i=1}^{3}\omega_{i}=1$; $H_{lp}\left({\hat{x}}_{ol}\left(1\right)\right)$, $H_{v}\left(v_{ol}\left(n\right)\right)$, and $H_{d}\left(L_{ol}\right)$ are the entropies of the last data sample, the variation rate, and the duration time of the current trend segment, respectively. The recommended value for $\omega_{i}$ is 1/3. According to the definition of entropy, $H_{lp}\left({\hat{x}}_{ol}\left(1\right)\right)$, $H_{v}\left(v_{ol}\left(n\right)\right)$, and $H_{d}\left(L_{ol}\right)$ can be calculated as

(30) $$\left\{\begin{array}{c} H_{lp}\left({\hat{x}}_{ol}\left(n\right)\right)=-p_{lp}\left({\hat{x}}_{ol}\left(n\right)\right)\log_{2}p_{lp}\left({\hat{x}}_{ol}\left(n\right)\right)\\ H_{v}\left(v_{o}l\left(n\right)\right)=-p_{v}\left(v_{ol}\left(n\right)\right)\log_{2}p_{v}\left(v_{ol}\left(n\right)\right)\\ H_{d}\left(L_{ol}\right)=-p_{d}\left(L_{ol}\right)\log_{2}p_{d}\left(L_{ol}\right) \end{array}\right.,$$

where $p_{lp}\left({\hat{x}}_{ol}\left(n\right)\right)$, $p_{v}\left(v_{ol}\left(n\right)\right)$, and $p_{d}\left(L_{ol}\right)$ denote the probabilities of samples in the distributions $F_{lp}$, $F_{v}$, and $F_{d}$ being larger than ${\hat{x}}_{ol}\left(n\right)$, $v_{ol}\left(n\right)$, and $L_{ol}$, respectively.

The mixture entropy in Equation (29) obtains a small value when the trend segment tends to arrive at the alarm threshold in a higher certainty. According to the two cases of a trend segment tending to arrive at the alarm threshold, its variation rate and variation time, or variation rate and amplitude value, should be different apparently from their values corresponding to the normal situations. Hence, the probabilities of the samples more than the variation rate, the variation time, and the amplitude value in their sample distributions are small, which results in small values of $H_{v}\left(v_{o}l\left(n\right)\right)$ and $H_{d}\left(L_{ol}\right)$, or $H_{lp}\left({\hat{x}}_{ol}\left(n\right)\right)$ and $H_{v}\left(v_{o}l\left(n\right)\right)$ in Equation (30). $H_{lp}\left({\hat{x}}_{ol}\left(n\right)\right)$, $H_{v}\left(v_{o}l\left(n\right)\right)$, and $H_{d}\left(L_{ol}\right)$ are the information entropy of the amplitude value, the variation rate, and the variation time, respectively. Hence, the trend segment tends to arrive at the alarm threshold corresponding to $H(x_{ol}\left(n\right))$ in Equation (29) having a small value resulting from small values of $H_{v}\left(v_{o}l\left(n\right)\right)$ and $H_{d}\left(L_{ol}\right)$, or $H_{lp}\left({\hat{x}}_{ol}\left(n\right)\right)$ and $H_{v}\left(v_{o}l\left(n\right)\right)$. To declare whether a trend segment tends to arrive at the alarm threshold, it is necessary to investigate a threshold for declaring whether $H(x_{ol}\left(n\right))$ in Equation (29) is small enough or not. Given the two cases of a trend segment most likely to arrive at the alarm threshold aforementioned, two heuristic thresholds for $H(x_{ol}\left(n\right))$ can be obtained as

(31) $$\left\{\begin{array}{c} H_{th,1}=-\omega_{2}P_{v,\epsilon_{2}}\log_{2}P_{v,\epsilon_{2}}-\omega_{3}P_{d,\epsilon_{3}}\log_{2}P_{d,\epsilon_{3}}\\ H_{th,2}=-\omega_{1}P_{lp,\epsilon_{1}}\log_{2}P_{lp,\epsilon_{1}}-\omega_{2}P_{v,\epsilon_{2}}\log_{2}P_{v,\epsilon_{2}} \end{array}\right..$$

Here, $P_{lp,\epsilon_{1}}$, $P_{v,\epsilon_{2}}$, and $P_{d,\epsilon_{3}}$ are the probabilities of ${\hat{x}}_{ol}\left(1\right)$, $v_{ol}\left(n\right)$, and $L_{ol}$ being more than their $100\times (1-\epsilon_{i})$ percent upper confidence limits, i=1,2,3, respectively; a typical value of the parameters $\epsilon_{i}$ is 0.05. It is clear that the values of $P_{lp,\epsilon_{1}}$, $P_{v,\epsilon_{2}}$, and $P_{d,\epsilon_{3}}$ are determined by their related $\epsilon_{i}$, and $H_{th,1}$ is equal to $H_{th,2}$ on the condition that $\omega_{i}$ and $\epsilon_{i}$ take the same values, respectively. Nonetheless, the threshold for declaring whether $H(x_{ol}\left(n\right))$ in Equation (29) is small enough or not can be selected in a general form as

(32) $$H_{th}=min\left\{H_{th,1},H_{th,2}\right\}.$$

With the optimal pre-warning threshold $r_{th,opt}$ in Equation (9) and the entropy threshold $H_{th}$ in Equation (32), the pre-warning data sequence $r_{w}\left(n\right)$ is generated by comparing $r_{ol}\left(n\right)$ in Equation (28) with $r_{th,opt}$ and comparing $H(x_{ol}\left(n\right))$ in Equation (29) with $H_{th}$ as

(33) $$r_{w}\left(n\right)=\left\{\begin{array}{cc} 1, & \mathrm{if}\;r_{ol}\left(n\right)\leq r_{th,opt}\wedge H\left(x_{ol}\left(n\right)\right)\leq H_{th}\\ 0, & \mathrm{otherwise} \end{array}\right..$$

### 3.4. Summary of the Proposed Method

The proposed pre-warning method is composed of an off-line design part and an online application part. In the off-line part, after extracting the last data samples, the variation rates, and the time durations of the trend segments contained in the historical data sequence through the PLR method, a sample distribution of the remaining time to alarm is obtained, and the optimal pre-warning threshold $r_{th,opt}$ is determined from this sample distribution through Bayesian estimation theory. In the online part, the last data sample, the variation rate, and the time duration of the current trend segment are extracted online to support the calculation of the remaining time to alarm and the mixture entropy, and a pre-warning is triggered when the remaining time to alarm is not longer than the optimal pre-warning threshold and the mixture entropy is small.

**Figure 3:**
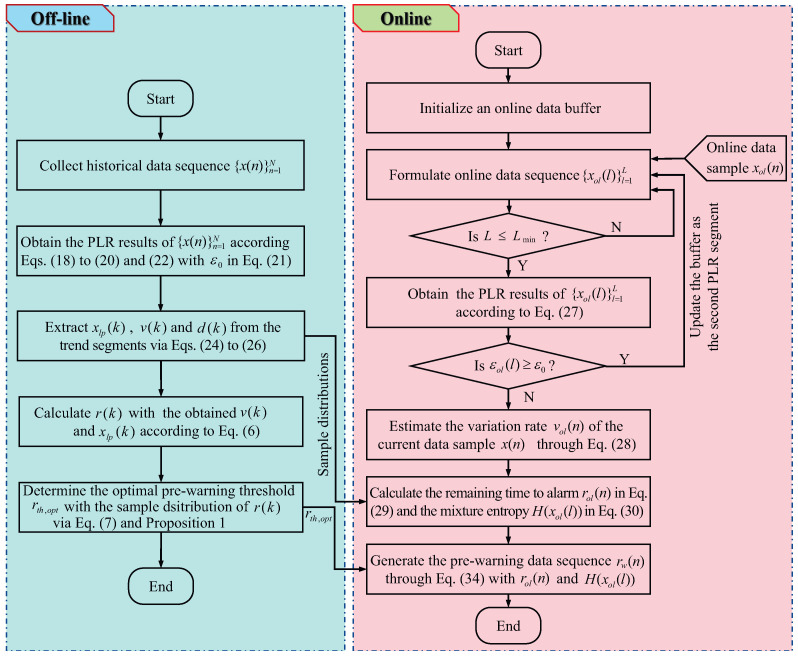
The flowchart of the proposed method.

The pseudo-code of the proposed pre-warning method is provided in Algorithm 1. The time and the space complexity of the online application part are analyzed as follows. The time complexity of the online application part is determined by the PLR algorithm, and the number of data samples in the online buffer is not larger than the buffer size *W*. By considering the time complexity of the PLR method [47], the time complexity of the online application part is O(W). The space cost of the online application part is mainly determined by the online buffer size and the PLR results of the data sequence in the online buffer, and, thus, the space complexity of the online application part is O(2W). The proposed method has been realized on a personal computer with an Intel Core i7-4770 CPU @3.40 GHz and 8 GB memory. The CPU is produced by the Intel Corporation, which is located in Santa Clara, CA, USA. The consuming time for an industrial data sample online is approximately 0.1644 s. To facilitate understanding, a flowchart of the proposed method is provided in Figure 3.
**Algorithm 1:** Pre-warning for the remaining time to alarm
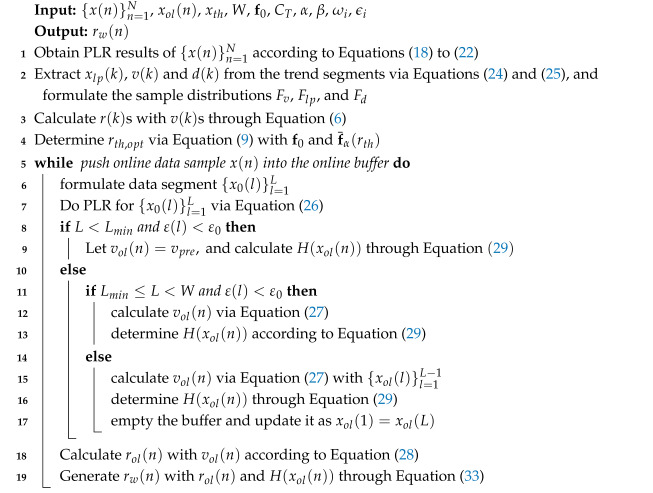


## 4. Examples

This section presents four numerical examples and an industrial example to show the effectiveness of the proposed method. The first numerical example is provided to illustrate the application procedures of the proposed method. The second numerical example is taken to verify the optimality of the determined optimal pre-warning threshold. The third numerical example is used to verify the necessity of the mixture entropy in pre-warning online. The fourth numerical example is available to make a comparison between the proposed method and a deep learning-based method. Finally, an industrial example is presented to show the effectiveness of the proposed method in practice.

### 4.1. Numerical Example A

This example is provided to verify the feasibility of the proposed method and to illustrate the application procedures. First, data sequences in normal situations were simulated to determine the optimal pre-warning threshold. Second, a data sequence in an abnormal situation was simulated to generate pre-warnings. All the simulated data sequences in the normal situations were generated with the variation rates following a normal distribution, while the abnormal data sequence was generated with a larger variation rate than its counterparts in the normal situations.

To simulate data sequences in normal situations, the basic un-noised data sequence was composed of an upward trend segment and a horizontal trend segment as

(34) $$x_{u}\left(n\right)\;=\;\left\{\begin{array}{c} 40,\mathrm{if}\;1\;\leq \;n\;\leq \;L_{u1}\\ 40\;+\;|b_{u}|\cdot \left(n\;-\;L_{u1}\;+\;1\right),\mathrm{if}\;L_{u1}+1\;\leq \;n\;\leq \;L_{u1}+L_{u2} \end{array}\right..$$

Here, $L_{u1}$ and $L_{u2}$ are the data lengths of $x_{u}\left(n\right)$ in different trends. $L_{u1}$ and $L_{u2}$ are random integers and take uniform distributions, i.e., $L_{u1}$∼U[100,200] and $L_{u2}$∼U[100,200]; $b_{u}$ denotes the variation rate of $x_{u}\left(n\right)$ in an upward trend, and $b_{u}$ follows a normal distribution with mean 0.01 and variance $6.25\times 10^{-6}$, that is, $b_{u}$∼$N(0.01,6.25\times 10^{-6})$. The basic data sequence was defined as a superposition of the basic un-noised data sequence in Equation (34) and a white noise sequence, i.e.,

(35) $$x\left(n\right)=x_{u}\left(n\right)+e\left(n\right),\;1\leq n\leq L_{u1}+L_{u2},$$

where e(n) is the Gaussian white noise with zero mean and standard deviation 0.01.

**Figure 4:**
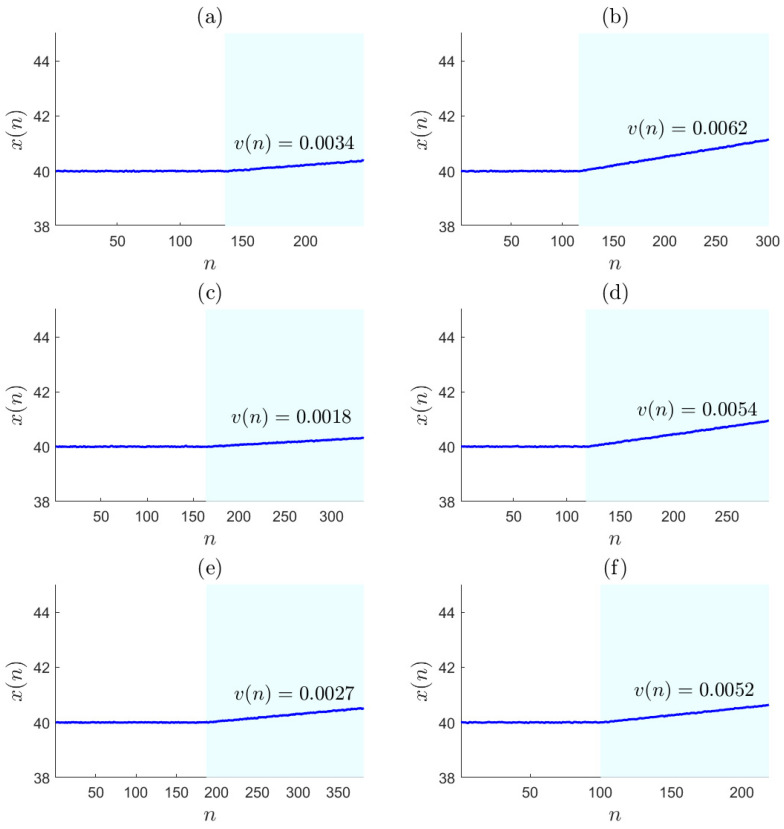
Six simulated basic data sequences (blue solid). (**a**) a simulated basic data sequence with the variation rate v(n)=0.0034 in its upward trend; (**b**) a simulated basic data sequence with the variation rate v(n)=0.0062 in its upward trend; (**c**) a simulated basic data sequence with the variation rate v(n)=0.0018 in its upward trend; (**d**) a simulated basic data sequence with the variation rate v(n)=0.0054 in its upward trend; (**e**) a simulated basic data sequence with the variation rate v(n)=0.0027 in its upward trend; (**f**) a simulated basic data sequence with its variation rate v(n)=0.0052 in the upward trend.

A simulated data sequence corresponding to normal situations was composed of 1000 basic data sequences in Equation (35) for determining the optimal pre-warning threshold $r_{th,opt}$. Six simulated basic data sequences are provided in Figure 4, in which the upward trends are marked with light-blue backgrounds. A high alarm threshold was selected for the simulation data sequence as $x_{th}=50$. The required false warning rate $f_{0}$ was set to 0.05, which is widely used as the probability of type **I** error. The PLR results of this simulated data sequence were obtained according to Equations (19)–(22) with $\varepsilon_{0}=0.0294$. The last data samples $x_{lp}\left(k\right)$s, the variation rates v(k)s, and the duration times d(k)s of all the trend segments were extracted from the PLR results, and the sample distributions $F_{v}$, $F_{lp}$, and $F_{d}$ were obtained, corresponding to $x_{lp}\left(k\right)$, v(k), and d(k), respectively.

With $x_{lp}\left(k\right)$ and v(k)s, the remaining time to alarm r(k)s was calculated via Equation (6). With the obtained r(k)s and the required false pre-warning rate $f_{0}=0.05$, the optimal pre-warning threshold was determined as $r_{th,opt}=541$ via Equation (9). Because the simulation data sequences were designed according to Equation (34), the real values of the variation rate v(k)s and the last data sample $x_{lp}\left(k\right)$s of each upward trend were known, so that the actual pre-warning threshold could be estimated from these values, and its value was 596. The optimal pre-warning threshold determined from the proposed method was very close to the actual pre-warning threshold, which clearly demonstrates the feasibility of the proposed method. To determine the threshold of the mixture entropy in Equation (32), we let the parameters $\omega_{i}=1/3$ and $\epsilon_{i}=0.05$, i=1,2,3, and $H_{th}=0.1406$ was obtained.

**Figure 5:**
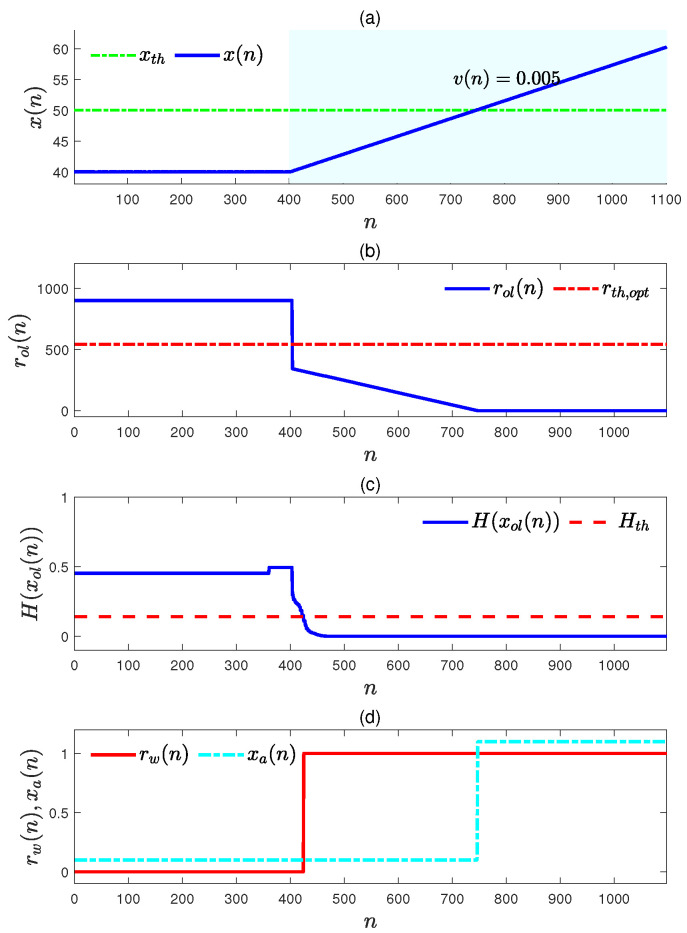
A simulated abnormal data sequence and its pre-warning sequence: (**a**) the simulated abnormal data sequence (blue solid) and the high alarm threshold $x_{th}=50$ (green dot–dash); (**b**) the sequence of the remaining time to alarm r(n) (blue solid) and the optimal pre-warning threshold $r_{th,opt}$ (red dot–dash); (**c**) the sequence of the mixture entropy $H(x_{ol}\left(n\right))$ (blue solid); (**d**) the sequence of pre-warning $r_{w}\left(n\right)$ (red solid) and the alarm data sequence $x_{a}\left(n\right)$ (cyan dot–dash).

To generate pre-warnings, a data sequence in an abnormal situation was simulated as shown in Figure 5a. The variation rate of the upward trend in this abnormal data sequence was 0.05, which is larger than the normal variation rates in Equation (34). The corresponding remaining time to alarm $r_{ol}\left(n\right)$ in Equation (28) could be obtained with $v_{ol}\left(n\right)$ in Equation (27), and the corresponding mixture entropy $H(x_{ol}\left(n\right))$ was calculated via Equation (29). The obtained sequence of $r_{ol}\left(n\right)$ is given in Figure 5b, and the mixture entropy is provided in Figure 5c. The pre-warning sequence $r_{w}\left(n\right)$ was generated based on $r_{ol}\left(n\right)$ and $H(x_{ol}\left(n\right))$ according to Equation (33), and this is shown in Figure 5d. As a comparison, the alarm data sequence $x_{a}\left(n\right)$ corresponding to the simulated abnormal data sequence is provided in Figure 5d also. It is obvious that the first sampling index of $r_{w}\left(n\right)=1$ is much smaller than the first sampling index of $x_{a}\left(n\right)=1$; that is, the proposed pre-warning method can perform its function as expected, and it can be concluded that the proposed method is feasible.

### 4.2. Numerical Example B

**Figure 6:**
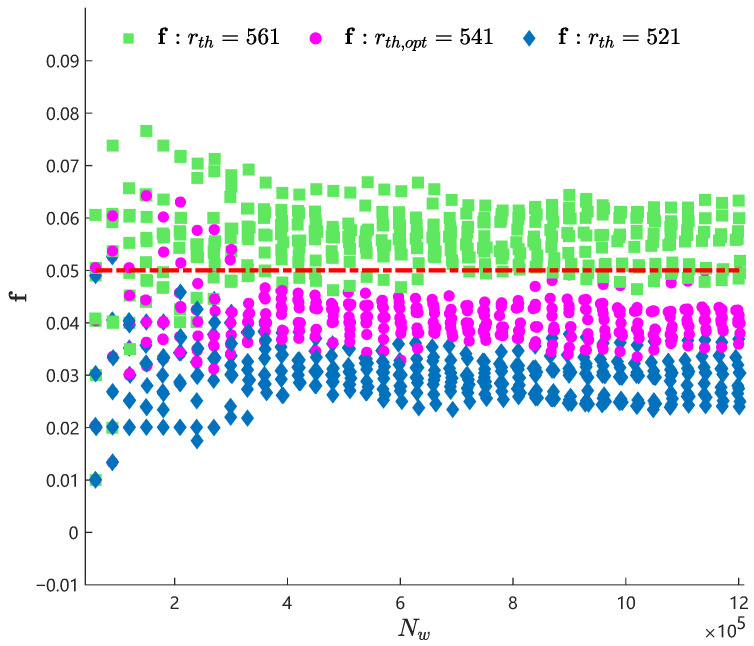
The false pre-warning rate fs obtained from ${\left\{x(n)\right\}}_{n=1}^{N_{w}}$ with $r_{th}=521$, $r_{th,opt}=541$, and $r_{th}=561$, and the required false pre-warning rate $f_{0}=0.05$ (red dot–dash).

This example verifies the optimality of the optimal pre-warning threshold. A group of new data sequences were simulated in normal situations according to Equation (35) to validate the optimality of the optimal pre-warning threshold $r_{th,opt}=541$ by checking whether its false warning rate was close to $f_{0}=0.05$ or not. The false warning rate used here is defined based on a pre-warning data sequence ${\left\{{\tilde{r}}_{w}\left(n\right)\right\}}_{n=1}^{N_{w}}$ and its related alarm remaining time sequence ${\left\{r_{ol}\left(n\right)\right\}}_{n=1}^{N_{w}}$ as

(36) $$f=\frac{C\left({\tilde{r}}_{w}\left(n-1\right)=1\right)\left|\right|{\tilde{r}}_{w}\left(n\right)=0)}{C(v_{ol}\left(n-1\right)\geq v_{th}||v_{ol}\left(n\right)<v_{th})},n\in \left[1,N_{w}\right],$$

where the operator C(·) obtains the number of the operand; ${\tilde{r}}_{w}\left(n\right)$ is a sample of the pre-warning data sequence and is obtained as

(37) $${\tilde{r}}_{w}\left(n\right)=\left\{\begin{array}{cc} 1, & \mathrm{if}\;r_{ol}\left(n\right)\leq r_{th,opt}\\ 0, & \mathrm{otherwise} \end{array}\right.,$$

the logical expression ${\tilde{r}}_{w}\left(n-1\right)=1\left|\right|{\tilde{r}}_{w}\left(n\right)=0$ denotes the instant of pre-warnings being triggered, and $v_{ol}\left(n-1\right)\geq v_{th}\left|\right|v_{ol}\left(n\right)<v_{th}$ indicates a trend segment of x(n) with a large positive variation rate. Here, ${\left\{r_{w}\left(n\right)\right\}}_{n=1}^{N_{w}}$ is obtained with a simulated normal data sequence ${\left\{x(n)\right\}}_{n=1}^{N_{w}}$, which is composed of a number of basic data sequences in Equation (35). Therefore, the simulated normal data length $N_{w}$ was a random variable. Note that the mixture entropy was not considered in Equation (37), due to the fact that the mixture entropy was not used in the selection process of the optimal pre-warning threshold. A group of false warning rates for ${\left\{x(n)\right\}}_{n=1}^{N_{w}}$ was calculated on the condition that the number of basic data sequences contained in ${\left\{x(n)\right\}}_{n=1}^{N_{w}}$ varied from 100 to 2000 with a step of 50. The false warning rates obtained from 10 independent groups of ${\left\{x(n)\right\}}_{n=1}^{N_{w}}$ are given in Figure 6 with magenta points. It can be observed that fs are located below $f_{0}$ with the length of the simulation data sequence increasing and convergence to a value that is very close to $f_{0}$.

The optimality of $r_{th,opt}=541$ was verified by comparing its false pre-warning rates with the false pre-warning rates of $r_{th}=521$ and $r_{th}=561$. The false pre-warning rates of $r_{th}=521$ and $r_{th}=561$ were calculated with the same simulation data sequences and in the same way. The calculated results are given in Figure 6 with green squares and cyan diamonds, respectively. Obviously, the false rates of $r_{th}=561$ were larger than $f_{0}$, and, hence, $r_{th}=561$ resulted in a large number of false pre-warnings. On the contrary, the false rates of $r_{th}=521$ were smaller than $f_{0}$, and there were much fewer false pre-warnings induced by $r_{th}=521$. Although the false pre-warning rates of $r_{th}=521$ were smaller than the false pre-warning rates of $r_{th,opt}=541$, the pre-warnings induced by $r_{th}=521$ had longer time delays than $r_{th,opt}=541$. The optimal pre-warning threshold yielded more reasonable false warning rates than the two other pre-warning thresholds. Thus, the optimality of the optimal pre-warning threshold was verified.

### 4.3. Numerical Example C

**Figure 7:**
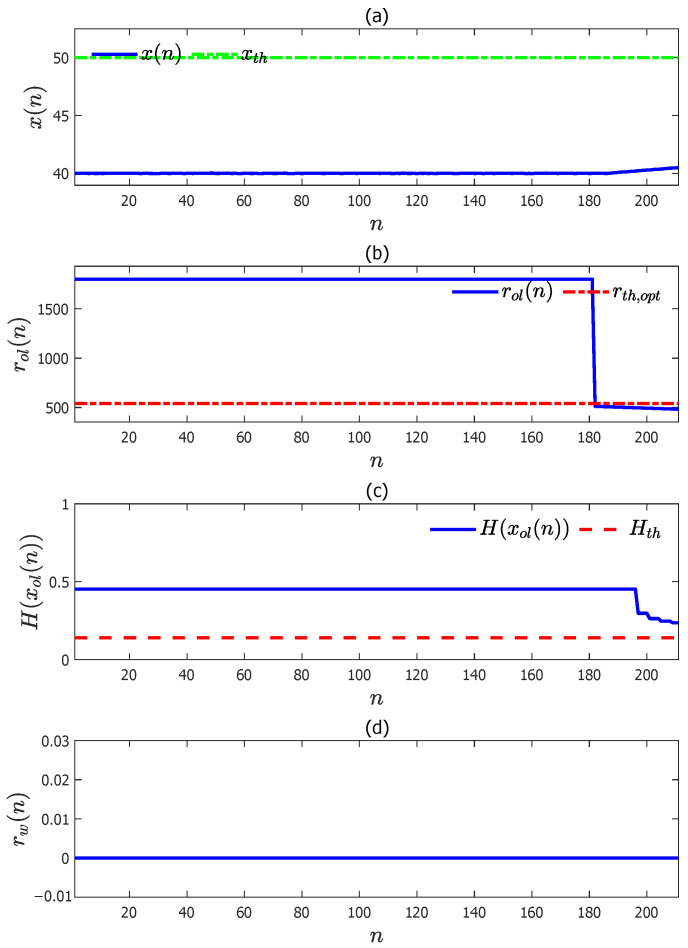
A simulated abnormal data sequence and its pre-warning sequence: (**a**) the simulated abnormal data sequence (blue solid) and the high alarm threshold $x_{th}=50$ (green dot–dash); (**b**) the sequence of the remaining time to alarm r(n) (blue solid) and the optimal pre-warning threshold $r_{th,opt}$ (red dot–dash); (**c**) the sequence of the mixture entropy $H(x_{ol}\left(n\right))$ (blue solid) and its threshold $H_{th}$ (red dash); (**d**) the sequence of pre-warnings $r_{w}\left(n\right)$ (blue solid).

The necessity of the mixture entropy in Equation (29) for generating pre-warnings online was verified in this case. An abnormal data sequence was designed to be similar to Equations (34) and (35). We let $b_{u}$ in Equation (34) be 0.02, and the simulated abnormal data sequence is given in Figure 7a. The abnormal data sequence contained an upward trend beginning at n=172 and ending at n=209.

To perform pre-warnings for this abnormal data sequence with the determined optimal pre-warning threshold $r_{th,opt}=541$, the remaining time to alarm data sequence $r_{ol}\left(n\right)$ was calculated according to Equation (28) with the variation rates estimated according to Equation (27), and it is presented in Figure 7b. It is obvious that $r_{ol}\left(n\right)$ was smaller than $r_{th,opt}=541$ when n≥182. If there was no mixture entropy to measure the certainty of the upward trend growing to the high alarm threshold (green dot–dash line) in Figure 7a then a false pre-warning was generated.

To avoid the false pre-warning generating pre-warnings online, the mixture entropy $H(x_{ol}\left(n\right))$ was calculated and plotted in Figure 7c (blue solid). It can be observed from Figure 7c that $H(x_{ol}\left(n\right))$ was always above the mixture entropy threshold $H_{th}=0.1406$ by taking $\omega_{i}=1/3$ and $\epsilon_{i}=0.05$, i=1,2,3. Finally, the pre-warning data sequence was generated according to Equation (33) with $r_{ol}\left(n\right)$ and $H(x_{ol}\left(n\right))$, and it is shown in Figure 7c with a red solid line. Obviously, there were no false pre-warnings. The correct pre-warning sequence benefited from the mixture entropy.

### 4.4. Numerical Example D

This example made a comparison between the proposed method and a deep learning-based method. By taking the remaining time to alarm as the feature to formulate pre-warnings, it was ready to adapt the deep learning-based method for the problem to be studied here. The deep learning-based method is based on the convolutional neural network–long short-term memory (CNN–LSTM) model for time series prediction [31].

To satisfy the data requirement in the CNN–LSTM model training, the un-noised data segments corresponding to abnormal situations were generated as

(38) $$x\left(n\right)\;=\;\left\{\begin{array}{c} 40+e(n),\mathrm{if}\;1\;\leq \;n\;\leq \;1000\\ 40\;+\;|g_{u}|\cdot \left(n\;-\;1\right)+e\left(n\right),\mathrm{if}\;1001\;\leq \;n\;\leq \;2200 \end{array}\right..$$

Here, $g_{u}$ is the variation rate and follows a normal distribution with mean zero and standard variance 0.03, that is, $g_{u}$∼$N(0,9\times 10^{-4})$; e(n) is the Gaussian white noise with zero mean and standard deviation 0.01. To indicate abnormal situations, all the variation rates $g_{u}$ in Equation (38) were selected as the ones larger than v(n)=0.005 in Figure 5a.

The CNN–LSTM model adopted here was composed of two convolution layers (32 filters therein) and four long short-term memory layers (128 filters therein), in addition to an input layer, an output layer, an exponential linear unit layer, a batch normalization layer, a sequence unfolding layer, and a dropout layer. To ensure the quality of the CNN–LSTM model, the maximum number of epochs for training was taken as 100, and a mini-batch with 1000 observations was adopted at each iteration. The CNN–LSTM model could be trained with a simulated data sequence and its corresponding sequence of remaining time to alarm, which was calculated according to Equation (6) with the un-noised data samples and real variation rates.

**Figure 8:**
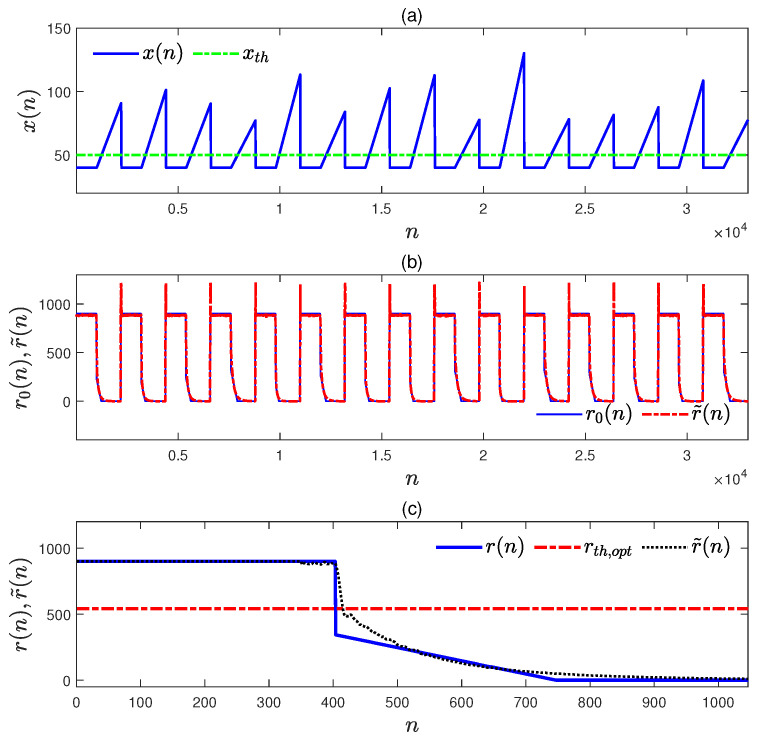
The test results of the CNN–LSTM model trained by a data sequence containing 100 abnormal data segments, and its application resulting in the abnormal data sequence in Figure 5a: (**a**) the test data sequence x(n); (**b**) the test data sequence $r_{0}\left(n\right)$ (blue solid) and the CNN–LSTM model predicated $\tilde{r}\left(n\right)$ (red dot–dash); (**c**) the sequences of r(n) and $\tilde{r}\left(n\right)$ for the abnormal data sequence in Figure 5a obtained from the proposed method (blue solid) and the CNN–LSTM model (black dotted).

For an ideal scenario, a simulated data sequence was generated to be composed of 100 abnormal data segments in Equation (38) and 100 normal data segments in Equation (34). The original data sequence and its corresponding sequence of remaining time to alarm were used to train and test an CNN–LSTM model. The proportions of the data used in training and testing were 85% and 15%, respectively. The test data sequence of x(n) and its corresponding sequence of remaining time to alarm $r_{0}\left(n\right)$ are provided in Figure 8a and b, separately. The trained model outputs $\tilde{r}\left(n\right)$ corresponding to the test data are provided in Figure 8b. It is obvious that $\tilde{r}\left(n\right)$ from the trained model can well describe the sequence $r_{0}\left(n\right)$. The sequence of remaining time to alarm is forecasted with the trained model for the abnormal data sequence in Figure 5a, and it is denoted by $\tilde{r}\left(n\right)$ in Figure 8c with the black dotted line. It is obvious that $\tilde{r}\left(n\right)$ can indicate the abnormal condition effectively.

**Figure 9:**
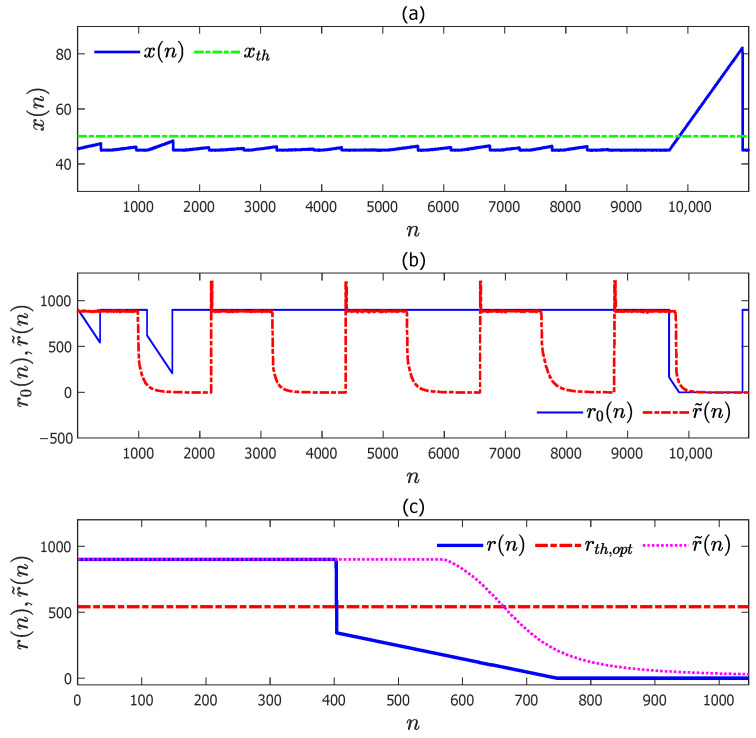
The test results of the CNN–LSTM model trained by a data sequence containing five abnormal data segments and its application result in the abnormal data sequence in Figure 5a: (**a**) the test data sequence x(n); (**b**) the test data sequence $r_{0}\left(n\right)$ (blue solid) and the trained CNN–LSTM model predicated $\tilde{r}\left(n\right)$ (red dot–dash); (**c**) the sequences of r(n) and $\tilde{r}\left(n\right)$ for the abnormal data sequence in Figure 5a obtained from the proposed method (blue solid) and the trained CNN–LSTM model (black dotted).

For the practical scenario, there are fewer abnormal data samples in industrial practice, and another CNN–LSTM model was trained with the data sequence composed of 5 abnormal data segments and 100 normal data segments. This CNN–LSTM model was trained in a similar manner as that in the ideal scenario. The testing results of this trained model are provided in Figure 9b, corresponding to the test data in Figure 9a. The sequence of remaining time to alarm was forecasted with this trained model for the abnormal data sequence in Figure 5a and is denoted by $\tilde{r}\left(n\right)$ in Figure 9c with the black dotted line. Clearly, $\tilde{r}\left(n\right)$ in Figure 9c had a long time delay to detect the abnormal condition.

By comparing the sequences of $\tilde{r}\left(n\right)$ and r(n) in Figure 8c and Figure 9c, it can be concluded that the deep learning-based method has the ability to predict the remaining time to alarm on the condition that a large number of abnormal data segments can be provided for training. However, such a condition is often not satisfied in industrial practice. On the contrary, the proposed method was not confined by this condition and had a better performance than the deep learning-based method in this practical scenario.

### 4.5. Industrial Example

The proposed method was applied to dozens of process variables in a large-scale thermal power plant. This industrial example was used to explain the necessity of the proposed pre-warning method and to illustrate the effectiveness and the feasibility of the proposed method.

**Figure 10:**
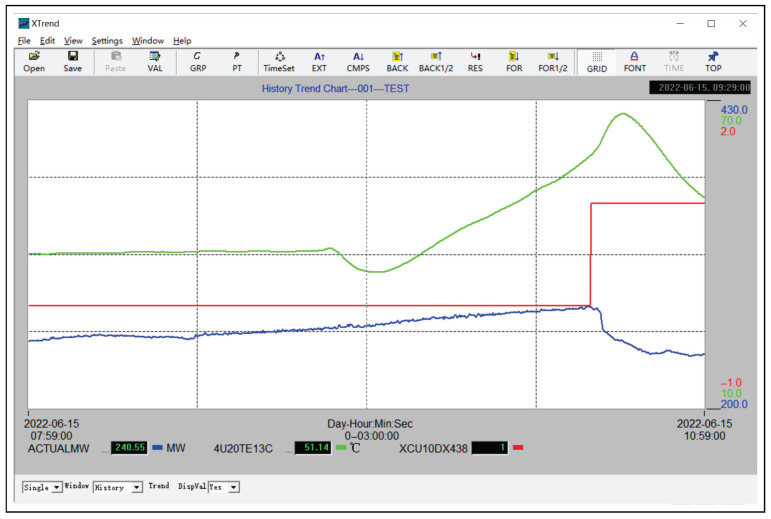
An accident in a large-scale thermal power generation plant.

An accident in a large-scale thermal power plant is provided in Figure 10, where data segments of three process variables with tagnames ACTUALM, 4U20TE13C, and XCU10DX438 are shown. The monitored process variable 4U20TE13C was the temperature of rotating machinery located in a coal mill, and its alarm threshold was configured as 50 °C (a high alarm threshold). As depicted in Figure 10, 4U20TE13C increased to its alarm threshold at approximately 9:34:00 and triggered an alarm at 10:20:54. This alarm caused the mill shutdown XCU10DX438 to switch on at 10:28:58, resulting in the desired active power ACTUALM decreasing by 25 MW. The time interval from the alarm being triggered to the mill shutdown was less than 15 min, which is the least time to start up a sparse mill. Clearly, the reason for the accident was that there was too little time left for the operators to start up a spare mill. Therefore, pre-warnings were necessary for 4U20TE13C.

A pre-warning was designed for 4U20TE13C, which is denoted as *x* for simplicity, and $x_{th}=50$ °C was its alarm threshold. A historical data sequence of 1 month was collected to determine the optimal pre-warning threshold $r_{th,opt}$. An accident data sequence in Figure 10 was taken to illustrate the pre-warning generated by the proposed method.

**Figure 11:**
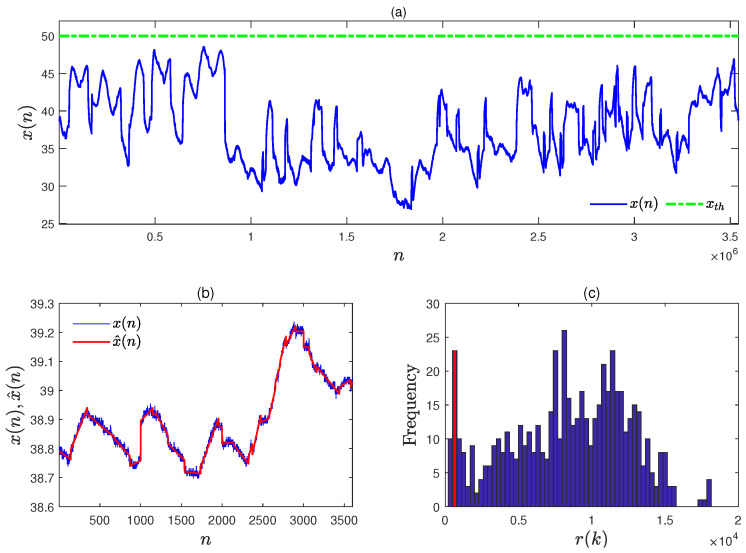
The industrial data sequence in normal situations and its related calculation results: (**a**) the collected industrial data sequence x(n) (blue solid) and its high alarm threshold $x_{th}$ (green dot–dash); (**b**) a subsegment of the industrial data sequence x(n) (blue solid) and its PLR results $\hat{x}\left(n\right)$ (red solid); (**c**) the obtained alarm remaining time r(k)s (blue bar) and the optimal pre-warning threshold $r_{th,opt}$ (red solid).

The optimal pre-warning threshold $r_{th,opt}$ in Equation (9) was determined with the historical data sequence lasting for 1 month. A subsegment of ${\left\{x\left(n\right)\right\}}_{n=1}^{2.678\times 10^{6}}$ is given in Figure 11a in the blue solid line, and the high alarm threshold $x_{th}$ is given in the green dot–dash line. It is worth noting that *x* is not a stable variable in a normal situation. Hence, it is not the same as traditional strategies to detect changes in stable variables. The PLR results of ${\left\{x\left(n\right)\right\}}_{n=1}^{2.678\times 10^{6}}$ were obtained from Equation (17) to Equation (22) with the separation threshold $\varepsilon_{0}=0.0301$, and some PLR results (the red solid lines) are provided in Figure 11b.

The trend segments with large enough variation rates were determined by comparing the variation rate v(k) in Equation (25) with the significant threshold of variation rate $v_{k,th}$ in Equation (23). That is, if the variation rate v(k) of a PLR data segment was larger than its significant threshold $v_{k,th}$, this PLR data segment had a large enough variation rate. The last data samples $x_{lp}\left(k\right)$s, the variation rates v(k)s, and the duration times d(k)s of all the trend segments were extracted from the PLR results, and the sample distributions $F_{v}$, $F_{lp}$, and $F_{d}$ were obtained in the meantime. Furthermore, the corresponding remaining time to alarm r(k)s was calculated via Equation (6) with the $x_{lp}\left(k\right)$s and v(k)s, and the r(k)s are provided in the histogram of Figure 11c. The optimal pre-warning threshold $r_{th,opt}$ was determined as $r_{th,opt}=568$ through Equations (9) and (10) by taking the false pre-warning rate $f_{0}=0.05$, and the location of $r_{th,opt}$ is marked with a red solid line in Figure 11c. Due to the fact that r(k)s are obtained from the historical data in normal situations, the false pre-warning rate $f_{0}$ is equivalent to the probability of type **I** errors, and $f_{0}=0.05$ is a common value for type **I** errors used in industrial practice [21,36]. In other words, $f_{0}=0.05$ indicates that there were 5% trend segments with large positive variation rates resulting in false pre-warnings.

**Figure 12:**
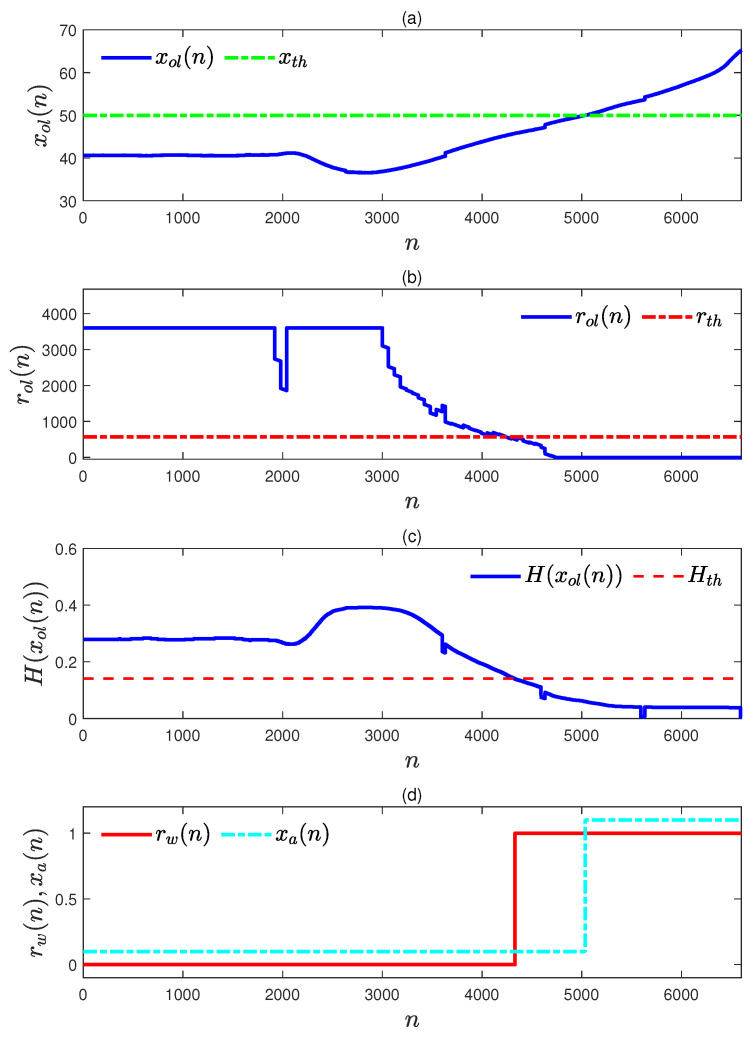
The accident data sequence and its related calculation results: (**a**) the accident data sequence $x_{ol}\left(n\right)$ and its alarm threshold $x_{th}$ (green dot–dash); (**b**) the sequence of alarm remaining time $r_{ol}\left(n\right)$ (blue solid) and the optimal pre-warning threshold $r_{th,opt}$ (red dot–dash); (**c**) the mixture entropy sequence H(xol(n)) for the accident data sequence $x_{ol}\left(n\right)$; (**d**) the obtained pre-warning sequence $r_{w}\left(n\right)$ (red solid) and the alarm data sequence $x_{a}\left(n\right)$ (cyan dot–dash) sequences.

By applying the proposed pre-warning method to the accident data sequence in Figure 10 with $r_{th,opt}=568$ in an online manner, an effective pre-warning was triggered. For the accident data sequence in Figure 12a (a part of x(n) in Figure 10), the remaining time to alarm sequence $r_{ol}\left(n\right)$s was obtained online through Equation (28), and the sequence of $r_{ol}\left(n\right)$ is plotted in Figure 12b. At the same time, the mixture entropy sequence of $H(x_{ol}\left(n\right))$ was calculated through Equations (31) and (29) with the obtained sample distributions of $F_{v}$, $F_{lp}$, and $F_{d}$. The pre-warning sequence $r_{w}\left(n\right)$ was calculated via Equation (33) and provided in Figure 12c. Obviously, for the abnormal data sequence in Figure 10 the optimal pre-warning threshold reflected the abnormal correctly and generated the desired pre-warning sequence $r_{w}\left(n\right)$. Therefore, the feasibility of the proposed method is illustrated and validated. As a comparison, the alarm data sequence $x_{a}\left(n\right)$ was generated according to Equation (5) and is provided with a cyan dash–dot line in Figure 12d. It can be observed that $r_{w}\left(n\right)=1$ occurred at n=4331 and that $x_{a}\left(n\right)$ switched into the alarm state at n=5036. Thus, the proposed pre-warning method can alert operators much earlier than traditional alarms and provides operators much more time to address the abnormality. Hence, the proposed method is beneficial for the safety of production processes.

In addition, the performance of the proposed method was validated with the historical data sequence in normal situations lasting for three months. Benefiting from the mixture entropy combined in Equation (33), the false pre-warning rates were much less than the required value $f_{0}=0.05$. The variation rates $v_{ol}\left(n\right)$ and the de-noised last sample ${\hat{x}}_{ol}\left(l\right)$ of the upward trends were obtained through the online PLR method. The online variation rate $v_{ol}\left(n\right)$ was calculated through Equation (27). The online remaining time to alarm $r_{ol}\left(n\right)$ was obtained with ${\hat{x}}_{ol}\left(n\right)$ and $v_{ol}\left(n\right)$ through Equation (28), and the pre-warning sequence $r_{w}\left(n\right)$ was generated via Equation (33) with $r_{ol}\left(n\right)$ and $r_{th,opt}=568$. There were 8, 11, and 7 false pre-warnings out of 291, 420, and 227 trend segments with large variation rates in the three months, respectively. Consequently, the false pre-warning rates for the three months were 2.7491%, 2.6190%, and 3.0837%, according to Equation (36). The false pre-warning rates were close to the required 5%. Therefore, it can be concluded that the proposed pre-warning method achieves a satisfactory performance, in terms of false pre-warnings.

## 5. Conclusions

This paper proposes a pre-warning method based on variation rates and mixture entropies for a special class of industrial process variables. In the off-line stage, by extracting the information of trend segments in the historical data sequence via PLR, the proposed method determines the optimal pre-warning threshold through Bayesian estimation theory with a sample distribution of the remaining time to alarm, and it formulates the sample distributions of the variation rates, the first data sample, and the duration time. In the online stage, the remaining time to alarm is estimated with the online-obtained variation rates and the last data sample of the current trend, and the mixture entropy is calculated with the variation rate, the first data sample, and the trend length of the current trend segment, as well as their sample distributions obtained in the off-line stage. The pre-warning sequence is generated on the condition that the remaining time to alarm is no longer than the optimal pre-warning threshold and the mixture entropy is small enough.

Although the proposed method achieves the desired performance, it could be developed in three aspects. First, to reduce the number of false pre-warnings, the proposed method could be incorporated with delay timers, alarm dead-bands, or their combinations that are effective in handling nuisance alarms [48]. However, such an incorporation would result in time delays for pre-warnings. A key issue is to achieve a good balance between the false pre-warning rate and the time delay for pre-warnings. Second, the pre-warning threshold could be designed as an adaptive one, in order to deal with non-stationary and nonlinear process variables. The key issues are to determine pre-warning thresholds corresponding to different normal situations and to detect the changes of these normal situations in an online manner. Third, to draw multivariate information from industrial processes, the proposed method could be extended to generate pre-warnings by exploiting normal operating zone models to describe the geometric space of an allowable variation region of multiple related variables [49]. A key issue is to extract the variation rates of multivariate data sequences in their corresponding high-dimensional geometric space.

## Data Availability

The data presented in this study are available on request from the corresponding author. The data are not publicly available, due to restrictions.

## Abbreviations

The following abbreviations are used in this manuscript:

PLR	piecewise linear representation
CNN–LSTM	convolutional neural network–long short-term memory
